# Reciprocal Interactions between Cell Adhesion Molecules of the Immunoglobulin Superfamily and the Cytoskeleton in Neurons

**DOI:** 10.3389/fcell.2016.00009

**Published:** 2016-02-16

**Authors:** Iryna Leshchyns'ka, Vladimir Sytnyk

**Affiliations:** School of Biotechnology and Biomolecular Sciences, The University of New South WalesSydney, NSW, Australia

**Keywords:** cell adhesion molecule, immunoglobulin superfamily, neural cell adhesion molecule, L1, cytoskeleton, neurons, neurite outgrowth, synapse

## Abstract

Cell adhesion molecules of the immunoglobulin superfamily (IgSF) including the neural cell adhesion molecule (NCAM) and members of the L1 family of neuronal cell adhesion molecules play important functions in the developing nervous system by regulating formation, growth and branching of neurites, and establishment of the synaptic contacts between neurons. In the mature brain, members of IgSF regulate synapse composition, function, and plasticity required for learning and memory. The intracellular domains of IgSF cell adhesion molecules interact with the components of the cytoskeleton including the submembrane actin-spectrin meshwork, actin microfilaments, and microtubules. In this review, we summarize current data indicating that interactions between IgSF cell adhesion molecules and the cytoskeleton are reciprocal, and that while IgSF cell adhesion molecules regulate the assembly of the cytoskeleton, the cytoskeleton plays an important role in regulation of the functions of IgSF cell adhesion molecules. Reciprocal interactions between NCAM and L1 family members and the cytoskeleton and their role in neuronal differentiation and synapse formation are discussed in detail.

## Immunoglobulin superfamily cell adhesion molecules (IgSF CAMs)

**Figure 1:**
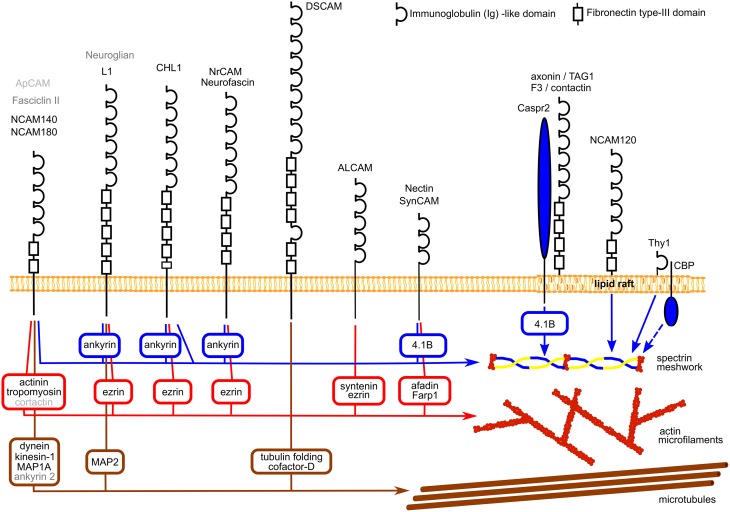
**Schematic diagram showing examples of the structure of IgSF CAMs and their links to the cytoskeleton components**. Linker proteins connecting IgSF CAMs to the cytoskeleton components are shown in square boxes. Note, that NrCAM and neurofascin, nectin and SynCAM, and axonin/TAG1 and F3/contactin have similar structures which are shown only once. Homologs of mammalian NCAM and L1 and their interaction partners in *Drosophila* and *Aplysia* are indicated in dark and light gray, respectively. See Table 1 for references.

IgSF CAMs are cell surface glycoproteins highly expressed in the developing and mature nervous system. They are characterized by a large extracellular domain containing one or several immunoglobulin-like (Ig) repeats (Shapiro et al., 2007). In a recent study, over 50 different members of this family were found to be expressed in the mammalian nervous system (Gu et al., 2015), however only some of them were extensively studied. While most of IgSF members are single-pass transmembrane proteins with intracellular domains of various lengths, some IgSF CAMs are anchored to the cell surface plasma membrane via a glycosylphosphatidylinositol (GPI) anchor (Figure 1).

The extracellular domains of IgSF CAMs typically mediate homophilic cell adhesion, i.e., cell adhesion mediated by interactions between the same molecules on membranes of adjacent cells, with Ig domains playing a key role in homophilic interactions (Zhao et al., 1998; Soroka et al., 2003; Shapiro et al., 2007; Kulahin et al., 2011). In addition, IgSF CAMs heterophilically interact with a variety of other cell surface receptors, cell adhesion molecules, and proteins of the extracellular matrix.

IgSF CAMs play key roles in the developing nervous system by regulating migration of neurons, growth and branching of axons and dendrites, and establishment of contacts between neurons (Dityatev et al., 2004; Dalva et al., 2007; Maness and Schachner, 2007; Hansen et al., 2008; Schmid and Maness, 2008). In the mature nervous system, these molecules play an essential role in the maintenance and plasticity of functionally important cell-to-cell contacts, such as synaptic contacts, specialized contacts between neurons mediating neurotransmission (Schachner, 1997; Gerrow and El-Husseini, 2006; Dityatev et al., 2008; Yang et al., 2014).

A number of IgSF CAMs expressed in neurons have been shown to interact with the neuronal cytoskeleton, which is composed of actin filaments, microtubules, and the submembrane actin-spectrin meshwork (Figure 1). While our knowledge about the complex picture of the multiple interactions between IgSF CAMs is still very fragmented, current evidence indicates that the relationship between IgSF CAMs and the neuronal cytoskeleton is reciprocal and that while IgSF members regulate the assembly of the cytoskeleton, the functioning of IgSF CAMs also depends on and is regulated by the cytoskeleton. In this review, we mostly focus on the reciprocal interactions between the cytoskeleton and the neural cell adhesion molecule (NCAM) and members of the L1 family including L1, close homologue of L1 (CHL1), neurofascin and neuronal-glial cell adhesion molecule related cell adhesion molecule (NrCAM) since interactions of these molecules and their homologs in invertebrates with the cytoskeleton have been extensively studied. Members of these families are highly expressed in the developing and mature nervous system. Multiple roles that NCAM and L1 family members play in regulation of neuronal development and function are the subject of a number of recent reviews (Maness and Schachner, 2007; Schmid and Maness, 2008; Kriebel et al., 2012; Sakurai, 2012; Schafer and Frotscher, 2012) and discussed only briefly here in the context of the interactions with the cytoskeleton. We also consider the evidence showing that reciprocal interactions with the cytoskeleton play an important role in functions of other members of IgSF as well.

## IgSF CAMs interact with the cytoskeleton components

Interactions with the cytoskeleton components have been described for a number of IgSF members expressed in the mammalian nervous system and also for their homologs in invertebrates (Figure 1; Table 1). Typically, binding to the cytoskeleton is mediated by the intracellular domains of IgSF members and can be either direct or via linker proteins (Figure 1). In the mammalian nervous system, the intracellular domains of two transmembrane isoforms of the neural cell adhesion molecule 1, NCAM140, and NCAM180, directly bind to βI spectrin (Leshchyns'ka et al., 2003). The intracellular domains of L1 family members contain a binding site for ankyrin, which links them to the spectrin meshwork (Garver et al., 1997; Hortsch et al., 1998a). The intracellular domains of nectins and nectin-like protein 2 (NECL-2), also called synaptic cell adhesion molecule (SynCAM)/cell adhesion molecule 1 (Cadm 1)/tumor suppressor in lung cancer 1 (TSLC-1), are connected to the actin cytoskeleton via linker proteins afadin and FERM, Rho/ArhGEF, and Pleckstrin domain protein 1 (Farp1), respectively (Takahashi et al., 1999; Cheadle and Biederer, 2012), while the intracellular domain of activated leukocyte cell adhesion molecule (ALCAM) is connected to the actin cytoskeleton via syntenin-1 and ezrin (Tudor et al., 2014). The intracellular domain of the Down syndrome cell adhesion molecule (DsCAM) is linked to tubulin via tubulin folding cofactor D (Okumura et al., 2015).

**Table 1:** **Examples of IgSF CAMs, which bind directly or indirectly via linker proteins to the cytoskeleton components**.

**Cell adhesion molecule**	**Cytoskeleton component (Reference, if the interaction was directly demonstrated)**	**Direct or proposed linker proteins (Reference where the interaction with the linker protein was shown)**
NCAM	Spectrin βI (Pollerberg et al., 1986; Leshchyns'ka et al., 2003)	Direct
	Tubulin (Buttner et al., 2003, 2005)	Dynein (Perlson et al., 2013) Kinesin-1 (Wobst et al., 2015) MAP 1A (Buttner et al., 2003)
	Actin	α-actinin, tropomyosin (Buttner et al., 2003)
Fasciclin II	Tubulin	Ankyrin 2 (long isoform; Pielage et al., 2008)
apCAM	Tubulin	Not shown (Lee and Suter, 2008)
	Actin	Cortactin (Decourt et al., 2009)
L1	Spectrin	Ankyrin B (Garver et al., 1997; Buhusi et al., 2008)
	Actin	Ezrin (Dickson et al., 2002)
	Tubulin	MAP2 (Poplawski et al., 2012)
Neuroglian	Spectrin	Ankyrin (Bouley et al., 2000; Enneking et al., 2013)
CHL1	βII spectrin (Tian et al., 2012)	Direct and via ankyrin (Buhusi et al., 2003)
	Actin	Ezrin (Schlatter et al., 2008)
Neurofascin	Spectrin	Ankyrin G (Garver et al., 1997; Tuvia et al., 1997; Zhang et al., 1998)
	Actin	Ezrin (Gunn-Moore et al., 2006)
NrCAM	Spectrin	Ankyrin (Davis and Bennett, 1994)
ALCAM	Actin	Syntenin-1, ezrin (Tudor et al., 2014)
DsCAM	Tubulin	Tubulin folding cofactor D (Okumura et al., 2015)
nectin	Actin	Afadin (Takahashi et al., 1999)
SynCAM 1	Actin	Farp1 (Cheadle and Biederer, 2012)
	Spectrin	Band 4.1-like protein 3, also called 4.1B (Yageta et al., 2002)

*Homologues of mammalian NCAM and L1 and their interaction partners in Drosophila and Aplysia are indicated in dark and light gray, respectively*.

**Figure 2:**
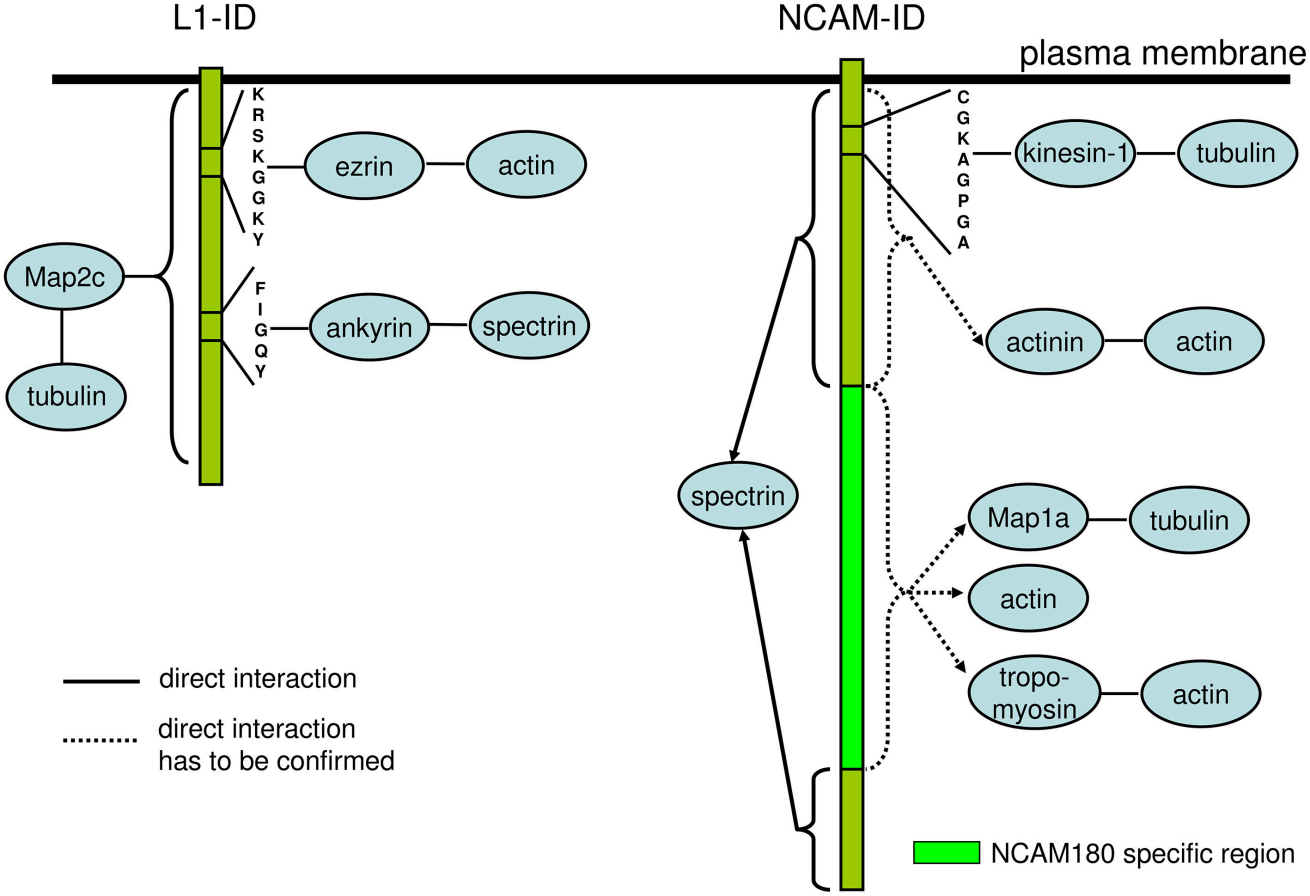
**Schematic diagram of the domains and motives within the intracellular domains of L1 and NCAM involved in interactions with the components of the cytoskeleton**. The intracellular domain of L1 (L1-ID) comprises binding motives for ezrin and ankyrin linking L1-ID to the actin and spectrin, respectively. MAP2c links L1-ID to tubulin, however, the binding sequence for MAP2c within L1-ID is not known. The intracellular domain of NCAM (NCAM-ID) comprises a binding motive for kinesin-1, which can link it to tubulin. This motive is present in both transmembrane isoforms of NCAM, NCAM140, and NCAM180, which contain the same amino acid sequences except for the presence of the NCAM180 specific region encoded by exon 18 in NCAM180. Intracellular domains of both transmembrane isoforms of NCAM directly bind to spectrin. The exact binding motive for spectrin is not known and is probably present in amino acid sequences present in both NCAM isoforms. Actinin, MAP1a, tropomyosin and actin were isolated from the brain using NCAM-ID as bait, however, the direct binding of these proteins has not been analyzed and exact binding sequences are not known. Since actinin binds to intracellular domains of NCAM140 and NCAM180, while MAP1a and tropomyosin bind to the intracellular domain of NCAM180 only, the binding sequences for these proteins are located in regions either present in both NCAM isoforms or in NCAM180 specific region, respectively. See Table 1 for references.

Interestingly, at least for some IgSF CAMs including NCAM and L1 family members, binding to multiple cytoskeleton components has been described with distinct domains within their intracellular domains being responsible for interactions with different cytoskeletal components. For instance, in addition to binding to βI spectrin, the intracellular domain of NCAM180 binds to tubulin and actin (Buttner et al., 2003; Figure 2). In addition to the ankyrin-binding site, the intracellular domain of L1 contains a binding site for ezrin, which links it to the actin cytoskeleton (Dickson et al., 2002). The intracellular domain of L1 can also bind directly to microtubule associated protein 2c (MAP2c), which can link it to tubulin (Poplawski et al., 2012; Figure 2). The intracellular domain of CHL1 contains a binding site for ezrin (Schlatter et al., 2008), and also directly binds to βII spectrin (Tian et al., 2012). These observations suggest that interactions of IgSF CAMs with the cytoskeleton can be amplified by multiple linker proteins, and that the intracellular domains of IgSF CAMs can function as scaffolds for the assembly of multiple cytoskeleton components. However, it is also possible that these interactions do not occur simultaneously, but are rather initiated in response to the changes in the subcellular localization or function of IgSF CAMs. This possibility is supported by observations showing that the interactions of IgSF CAMs with the cytoskeleton are highly regulated. For example, the interaction of L1 with ankyrin is regulated by phosphorylation in response to the extracellular signals (Garver et al., 1997), while the CHL1/βII spectrin complex is disrupted in response to Ca^2+^ influx induced by the binding of CHL1 to its ligands (Tian et al., 2012).

Interactions of IgSF CAMs with the cytoskeleton have also been described for the homologs of the mammalian IgSF CAMs in invertebrates. For example, in a sea slug *Aplysia*, a cell adhesion molecule apCAM, a structural homologue of the mammalian NCAM, associates with actin microfilaments and microtubules via linker proteins, such as cortactin (Suter et al., 1998; Lee and Suter, 2008; Decourt et al., 2009). In a fruit fly *Drosophila*, a cell adhesion molecule fasciclin II, also a structural homologue of the mammalian NCAM, is linked to the microtubules via linker proteins including a long isoform of ankyrin 2 (Pielage et al., 2008). The intracellular domain of neuroglian, a *Drosophila* homologue of mammalian L1, binds to *Drosophila* homologue of ankyrin (Hortsch et al., 1998b). Interestingly, the intracellular domain of human L1 is also able to bind to the *Drosophila* homologue of ankyrin (Hortsch et al., 1998b). Thus interactions between IgSF CAMs and the cytoskeleton components appear as highly evolutionary conserved.

Interestingly, interactions with the cytoskeleton have been described not only for transmembrane IgSF CAMs but also for the members of this superfamily which do not contain the intracellular domain but are linked to the plasma membrane via a GPI-anchor. For example, βI spectrin co-immunoprecipitates with a GPI-anchored isoform of mouse NCAM, NCAM120 (Leshchyns'ka et al., 2003) in accordance with previous reports showing that a large fraction of NCAM120 is immobile or shows restricted mobility in the cell surface plasma membranes (Simson et al., 1998). The interaction between NCAM120 and βI spectrin is disrupted after depletion of the cellular cholesterol (Leshchyns'ka et al., 2003). Similarly, transient anchorage of a mouse GPI-anchored IgSF CAM Thy-1 to the cytoskeleton is abolished by cholesterol depletion (Chen et al., 2006). In addition, transient anchorage of mouse Thy-1 to the cytoskeleton depends on the transmembrane protein Csk-binding protein (CBP; Chen et al., 2009). Therefore, interactions with lipids and transmembrane proteins appear to play key roles in linking GPI-anchored IgSF members to the cytoskeleton.

## IgSF CAMs regulate the assembly of the submembrane cytoskeleton

**Figure 3:**
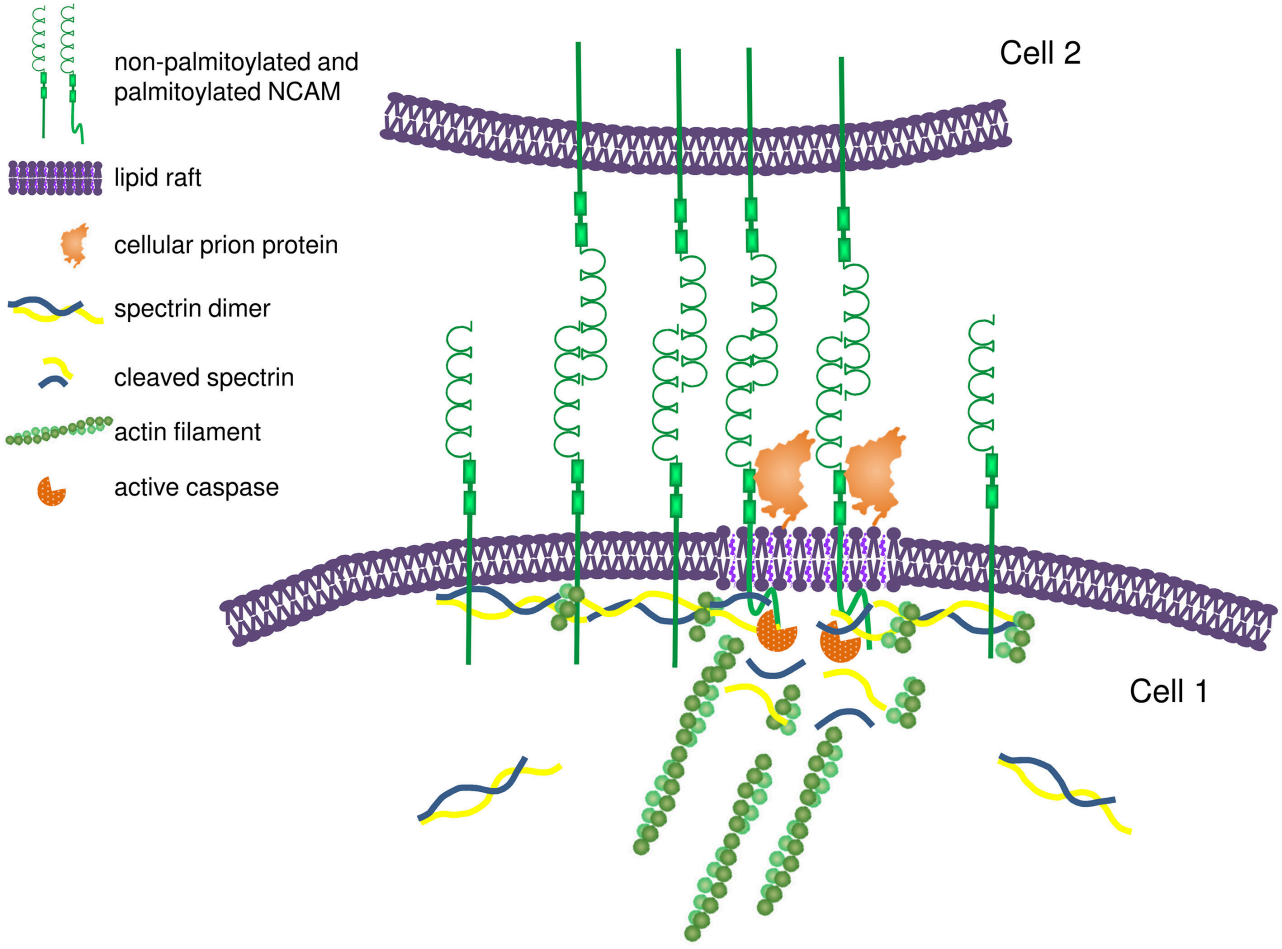
**Proposed model for NCAM-dependent assembly and remodeling of the submembrane spectrin-actin cytoskeleton**. Homophilic interactions between NCAM molecules on adjacent membranes promote clustering of NCAM at interneuronal contacts. The intracellular domains of NCAM bind to spectrin and promote its recruitment to the cell surface plasma membrane, thereby inducing formation of the spectrin-actin meshwork. Formation of the spectrin-actin meshwork results in further clustering of NCAM by limiting its diffusion in addition to the effect of homophilic interactions. Clustering of NCAM promotes partitioning of a subpopulation of NCAM molecules into lipid rafts due to interactions between the extracellular domain of NCAM and prion protein and palmitoylation of the intracellular domain of NCAM. In lipid rafts, NCAM promotes recruitment and activation of caspases, which induce partial local proteolysis of the spectrin-actin meshwork. The release of the short actin filaments from the spectrin-actin meshwork provides nucleation sites for formation and remodeling of the actin filaments.

A number of observations indicate that IgSF CAMs are directly involved in regulation of the assembly of the submembrane cytoskeleton. In cultured mouse hippocampal neurons and Chinese hamster ovary (CHO) cells used as model system, overexpression of NCAM results in an increase in the levels of polymerized βI spectrin indicating that NCAM not only binds to but also promotes polymerization of the βI spectrin meshwork beneath the cell surface plasma membrane (Leshchyns'ka et al., 2003; Figure 3). In an African green monkey kidney fibroblast-like COS7 cell line, overexpressed NCAM180 promotes capture and tethering of the microtubule plus-ends at the cell surface by binding to dynein, which binds to the intracellular domain of NCAM180 and links it to microtubules (Perlson et al., 2013). NCAM180-dependent cell adhesion is abolished after disruption of the association of NCAM180 with dynein, indicating that the association with the cytoskeleton is required (Perlson et al., 2013). In human embryonic kidney HEK293 cells, overexpression of CHL1 results in the recruitment of ankyrin to the cell surface plasma membrane (Buhusi et al., 2003). Clustering of two other members of the L1 family, neurofascin and NrCAM, at nodes of Ranvier in rats precedes redistribution of ankyrin G, also suggesting that these cell adhesion molecules define the initial site for subsequent assembly of the ankyrin-containing spectrin meshwork (Lambert et al., 1997). In primary rat fibroblasts, endogenous, or heterologous Thy-1 expression promotes focal adhesion and stress fiber formation by modulating the activity of p190 RhoGAP and Rho GTPase (Barker et al., 2004), indicating that GPI-anchored IgSF CAMs also play a role in the cytoskeleton regulation.

The importance of the interactions between IgSF CAMs and the cytoskeleton for the assembly of the cytoskeleton in functionally important subcellular compartments is also demonstrated by the analysis of neuronal synapses. Spectrin βI is one of the major components of the post-synaptic cytoskeleton in synapses. Synaptic levels of βI spectrin are reduced in synapses formed by cultured hippocampal neurons from NCAM-deficient mice and in synaptosomes isolated from brains of NCAM deficient mice indicating that NCAM regulates synaptic targeting of spectrin (Sytnyk et al., 2006). Disruption of the interaction between NCAM180 and dynein also perturbs preferential microtubule plus-end dynamics at synapses of cultured cortical neurons (Perlson et al., 2013). The intracellular domain of SynCAM 1 binds to Farp1, which regulates synaptic actin cytoskeleton polymerization via Rac1. Levels of Farp1 and polymerized actin are reduced in synapses of SynCAM 1 knockout mice indicating that SynCAM 1 is required for Farp1 recruitment to synaptic membranes (Cheadle and Biederer, 2012).

Studies in mammalian cells are consistent with observations in *Drosophila* showing that the recruitment of ankyrin to cell-cell contacts in *Drosophila* S2 cells is dependent on neuroglian-mediated cell adhesion (Hortsch et al., 1998b) and is required for neuroglian-mediated cell adhesion (Hortsch et al., 1998a). Furthermore, the homophilic interaction between GPI-anchored human TAG-1 and human L1 overexpressed in *Drosophila* S2 cells results in TAG-1-dependent recruitment of *Drosophila* ankyrin to areas of cell contacts (Malhotra et al., 1998) again indicating that the function of IgSF CAMs in regulation of the cytoskeleton is highly conserved during evolution. In *Drosophila* synapses, neuroglian has been shown to play a role in anchoring of microtubules in synapses possibly via interaction with a microtubule stabilizing protein doublecortin (Godenschwege et al., 2006) and giant ankyrin, a long isoform of *Drosophila* ankyrin 2 (Pielage et al., 2008).

Interestingly, several studies indicate that IgSF members also play a role in transcriptional and post-translational regulation of the expression of the cytoskeleton components they bind to. For example, overexpression of NCAM results in overall increased levels of βI spectrin in cultured mouse hippocampal neurons and CHO cells while levels of βI spectrin are reduced in brains of NCAM deficient mice (Leshchyns'ka et al., 2003). The expression of MAP2c in the mouse brain is regulated by L1 both at the protein and mRNA levels (Poplawski et al., 2012). In *Drosophila*, protein but not mRNA levels of neuronal ankyrin are reduced in neuroglian null mutants (Bouley et al., 2000). Thus, it is possible that IgSF members not only promote the assembly of the cytoskeleton components at the functionally important domains at the plasma membrane but also regulate the expression of the key cytoskeletal components and regulators.

## Ligand-induced remodeling of the cytoskeleton by IgSF CAMs

Binding of IgSF members to their extracellular ligands induces a plethora of the intracellular signaling cascades converging on enzymes involved in the cytoskeleton regulation (Maness and Schachner, 2007; Hansen et al., 2008; Schmid and Maness, 2008). In agreement, a number of observations indicate that binding of IgSF CAMs to their ligands in the extracellular environment results in changes in the structure of the cytoskeleton. These changes include redistribution of the cytoskeleton components to the sites of interactions of IgSF CAMs and changes in the level of polymerization of the cytoskeleton components.

In mammalian cells, ligand-induced clustering of NCAM induces accumulation of polymerized detergent insoluble βI spectrin in NCAM clusters (Sytnyk et al., 2006; Figure 3). In addition, clustering of NCAM also increases levels of the proteolytic cleavage products of βI spectrin via activation of caspases-8 and -3 and local and partial proteolysis of the components of the spectrin meshwork (Westphal et al., 2010; Figure 3). While the molecular mechanisms of the coordinated assembly and remodeling of the cytoskeleton by NCAM remain incompletely understood, cholesterol-enriched lipid microdomains, called lipid rafts, may play a significant role. A small portion of the transmembrane isoforms of NCAM is associated with lipid rafts, and this association is enhanced in response to clustering of NCAM by its ligands inducing palmitoylation of the intracellular domain of NCAM and interactions with the GPI-anchored cellular prion protein (Niethammer et al., 2002; Bodrikov et al., 2005; Santuccione et al., 2005). Lipid rafts are enriched in initiator caspase-8, a caspase-3 activator (Westphal et al., 2010). The association of caspase-8 and -3 with lipid rafts is reduced in NCAM deficient neurons, indicating that NCAM is important for targeting of caspases to these signaling platforms to induce remodeling of the spectrin cytoskeleton (Westphal et al., 2010). The release of short actin filaments via partial proteolysis of the spectrin meshwork can provide nucleation sites for polymerization of the new microfilaments (Figure 3). It is interesting in this respect that NCAM also induces the lipid raft-dependent activation of p21-activated kinase 1 (Pak1) and its downstream effectors LIMK1 and cofilin, which are involved in the regulation of the assembly of the actin microfilaments (Li et al., 2013). Levels of the filamentous actin are reduced in the brains of NCAM-deficient mice and filopodium motility is reduced in developing NCAM-deficient neurons indicating that NCAM-dependent cytoskeleton remodeling is functionally important (Li et al., 2013).

Ligand binding by the mammalian L1 family members L1, CHL1, NrCAM, and neurofascin also induces their association with lipid rafts (Ren and Bennett, 1998; Falk et al., 2004; Tian et al., 2012). Levels of polymerized ankyrin-B/βII spectrin are increased in CHL1 deficient mouse brains. Ligand induced clustering of CHL1 in wild type cultured mouse neurons induces partial removal of βII spectrin from the plasma membrane and internalization of CHL1 in a lipid raft-dependent manner indicating that CHL1 also plays a role in the remodeling of the spectrin meshwork (Tian et al., 2012).

The role of the IgSF CAM-ligand interactions in cytoskeleton remodeling is also supported by the work in *Aplysia* showing dynamic rearrangements of the cytoskeleton in growth cones of the growing neurites in response to ligands of apCAM. Clustering of apCAM at the cell surface of growth cones using beads covered with antibodies against apCAM or with purified native apCAM results in the coupling of the beads to the retrograde actin flow (Suter et al., 1998). Immobilization of the beads induces the restructuring of the actin filaments followed by the extension of the microtubules toward the immobilized apCAM accumulations (Suter et al., 1998, 2004). Interestingly, coupling of apCAM to the cytoskeleton is regulated by the src family tyrosine kinases, which accumulate in lipid rafts, suggesting that lipid rafts also play a role in apCAM-dependent cytoskeleton remodeling (Suter and Forscher, 2001).

## The cytoskeleton regulates the functions of IgSF CAMs

A number of observations indicate that while IgSF CAMs regulate the cytoskeleton, the cytoskeleton plays a direct role in regulation of the functions of cell adhesion molecules of this superfamily primarily by regulating the levels of these proteins at functionally important domains of the cell surface plasma membrane of neurons.

In mammalian cells, interactions with the βI spectrin meshwork reduce the lateral diffusion in the cell surface plasma membrane of NCAM, and particularly its largest isoform NCAM180 with the longest intracellular domain (Pollerberg et al., 1986). This reduction in the mobility is required for the accumulation of NCAM180 at the post-synaptic sites of excitatory synapses in cultured mouse hippocampal neurons (Leshchyns'ka et al., 2011). Similarly, depolymerization of the actin cytoskeleton using latrunculin A induces dispersion of Nectin-1, an IgSF cell adhesion molecule which interacts with actin via afadin (Takahashi et al., 1999), from synapses of developing neurons accompanied by the shedding of Nectin-1 from the cell surface (Lim et al., 2008).

The lateral mobility of neurofascin in the cell surface plasma membrane of B104 rat neuroblastoma cells is reduced by interactions with ankyrin, which links it to the spectrin meshwork (Garver et al., 1997). Interestingly, binding to ankyrin is required for neurofascin-mediated cell adhesion (Tuvia et al., 1997) indicating that the cytoskeleton is directly involved in regulation of the functions of this cell adhesion molecule. The ankyrin-binding motive of neurofascin is also required for tethering of neurofascin in the axonal initial segment in cultured rat hippocampal neurons (Boiko et al., 2007). Interactions with the actin cytoskeleton also reduce the mobility of L1 and Thy-1 at the initial segment of the axon in cultured rat hippocampal neurons, which is required for the maintenance of the polarized distribution of these proteins in neurons (Winckler et al., 1999). Interactions of TAG-1 with protein 4.1B via transmembrane Caspr2 are important for the association of the GPI-anchored TAG-1 with the spectrin meshwork and accumulation of TAG-1 at juxtaparanodes in myelinated axons (Cifuentes-Diaz et al., 2011). Also in *Drosophila*, the lateral mobility of neuroglian is regulated by interactions with ankyrin 2 (Enneking et al., 2013).

The spectrin meshwork not only reduces the mobility, but also regulates the removal of L1 family members from the neuronal cell surface by endocytosis. Disruption of the interaction between L1 and ankyrin in rat B35 neuroblastoma cells by pathogenic mutations within the ankyrin binding site results in increased endocytosis of L1 (Needham et al., 2001). Furthermore, knock-down of αII spectrin expression using siRNA interference results in reduced levels of L1 at the cell surface and reduced clustering of L1 in growth cones in human neuroblastoma SH-SY5Y cells (Trinh-Trang-Tan et al., 2014). Similarly, disruption of the interaction between CHL1 and βII spectrin by knock-down of βII spectrin expression using targeted siRNA results in increased endocytosis of CHL1 in cultured mouse hippocampal neurons (Tian et al., 2012).

Other components of the cytoskeleton play an indirect role in regulation of the cell surface levels of cell adhesion molecules by being involved in their intracellular transport. The intracellular domain of NCAM binds to a microtubule-binding molecular motor kinesin-1, which promotes the delivery of NCAM to the cells surface (Wobst et al., 2015), while kinesin-4 plays a role in the transport of L1 (Peretti et al., 2000). It remains to be investigated whether microtubules and microfilaments are also directly involved in regulation of the cell surface distribution of IgSF CAMs.

In addition to regulation of the cell surface distribution of IgSF CAMs, the cytoskeleton components play a key role in linking IgSF CAMs to a number of other proteins, such as other receptors, enzymes and ion channels required for signal transduction across the plasma membrane in response to binding of IgSF members to their ligands in the extracellular environment. For example, βI spectrin links NCAM to the receptor protein tyrosine phosphatase α (RPTPα), N-methyl-D-aspartate (NMDA) receptors, and intracellular enzymes including protein kinase C (PKC) and Ca^2+^/calmodulin-dependent protein kinase II (CaMKII) (Leshchyns'ka et al., 2003; Bodrikov et al., 2005, 2008; Sytnyk et al., 2006). Interactions of L1 with ankyrin B are also required for L1-dependent intracellular signaling including elevation of cyclic AMP levels in neurons (Ooashi and Kamiguchi, 2009).

## The role of the cytoskeleton in IgSF CAM – dependent neuronal differentiation and synapse formation

Current data indicates that the cytoskeleton plays a direct role in IgSF CAM-mediated functions. Soluble and substrate-immobilized ligands of IgSF CAMs, including antibodies against the extracellular domains of IgSF CAMs or recombinant fragments of the extracellular domains of IgSF CAMs, trigger changes in the interactions between IgSF CAMs and the cytoskeleton and also function as potent regulators of neuronal differentiation and function. For example, application of recombinant extracellular domains of L1 triggers interactions between the intracellular domain of L1 and ankyrin B and induces neurite initiation in cultured mouse cerebellar granule cells (Nishimura et al., 2003). Application of antibodies against the extracellular domain of NCAM triggers interactions of NCAM with spectrin (Sytnyk et al., 2006) and induces neurite outgrowth in cultured mouse hippocampal neurons (Chernyshova et al., 2011). Clustering of NrCAM using microspheres coated with its ligands, recombinant NrCAM, or antibodies against NrCAM, induces linkage between NrCAM and the F-actin retrograde flow required for NrCAM-dependent regulation of neurite outgrowth in cultured mouse cerebellar granule cells (Faivre-Sarrailh et al., 1999).

The functional role of the interactions between the cytoskeleton and IgSF CAMs is supported by studies showing that IgSF CAM-mediated neuronal differentiation and synapse formation are inhibited when these interactions are disrupted. In developing cultured mouse hippocampal neurons, disruption of the association between NCAM and βI spectrin using dominant-negative spectrin fragments results in inhibition of NCAM-dependent neurite outgrowth (Leshchyns'ka et al., 2003). L1-mediated branching in developing cultured mouse cerebellar neurons is inhibited by deletion of the binding sites for ezrin, which links L1 to the actin cytoskeleton (Cheng et al., 2005). Disruption of the binding of CHL1 to ankyrin abolishes CHL1-dependent cell migration in HEK293 cells (Buhusi et al., 2003). In mature cultured hippocampal and cortical neurons, disruption of the interaction between NCAM and βI spectrin or NCAM and dynein results in reduced formation of synapses (Sytnyk et al., 2006; Perlson et al., 2013). Spectrin cytoskeleton-enriched postsynaptic densities are thinner in synapses of NCAM-deficient hippocampal neurons and contain increased numbers of perforations (Puchkov et al., 2011). These defects are accompanied by increased endocytosis of the postsynaptic glutamate receptors of the AMPA type via perforations, indicating that the NCAM-dependent assembly of the βI spectrin meshwork is functionally important for the maintenance of the protein composition of the postsynaptic density (Puchkov et al., 2011). Mutations disrupting the interactions between neuroglian and ankyrin in *Drosophila* result in impaired synapse stability and abnormal neuromuscular junction growth (Enneking et al., 2013).

Importantly, pathogenic mutations in the ankyrin-binding site of the intracellular domain of L1 in humans with the X-linked mental retardation syndrome CRASH (corpus callosum hypoplasia, retardation, aphasia, spastic paraplegia, hydrocephalus) have been shown to reduce the interaction between L1 and ankyrin indicating that disruptions in the association with the cytoskeleton can directly contribute to the mechanisms of neuronal dysfunction in human X-linked mental retardation (Needham et al., 2001; Vos and Hofstra, 2010). Changes in the protein levels of L1 and other IgSF CAMs associated either with single nucleotide polymorphisms, environmental factors or pathological processes are found in a number of other conditions including *Alzheimer's* disease and psychiatric disorders (Atz et al., 2007; Gibbons et al., 2009; Gray et al., 2010; Leshchyns'ka et al., 2015). Whether these changes affect the bidirectional relationship between IgSF CAMs and the cytoskeleton and whether they contribute to the etiology of brain disorders are important questions for future studies.

## Author contributions

All authors listed, have made substantial, direct, and intellectual contribution to the work, and approved it for publication.

### Conflict of interest statement

The authors declare that the research was conducted in the absence of any commercial or financial relationships that could be construed as a potential conflict of interest.
